# Alterations in inflammatory markers after a 12-week exercise program in individuals with schizophrenia—a randomized controlled trial

**DOI:** 10.3389/fpsyt.2023.1175171

**Published:** 2023-05-11

**Authors:** Therese Torgersen Bigseth, John Abel Engh, Eivind Andersen, Gry Bang-Kittilsen, Jens Egeland, Ragnhild Sørum Falk, Tom Langerud Holmen, Jon Mordal, Jimmi Nielsen, Thor Ueland, Torkel Vang, Mats Fredriksen

**Affiliations:** ^1^Institute of Clinical Medicine, Faculty of Health Sciences, University of Oslo, Oslo, Norway; ^2^Division of Mental Health and Addiction, Vestfold Hospital Trust, Tønsberg, Norway; ^3^Faculty of Humanities, Sports and Educational Science, University of Southeast Norway, Horten, Norway; ^4^Department of Psychology, University of Oslo, Oslo, Norway; ^5^Oslo Centre for Biostatistics and Epidemiology, Research Support Services, Oslo University Hospital, Oslo, Norway; ^6^Mental Health Centre Glostrup, Copenhagen University Hospital, Copenhagen, Denmark; ^7^Research Institute of Internal Medicine, Oslo University Hospital Rikshospitalet, Oslo, Norway; ^8^K.G. Jebsen TREC, University of Tromso, Tromso, Norway

**Keywords:** schizophrenia, exercise, inflammation, inflammatory marker, suPAR, CRP, TNF, IL-6

## Abstract

**Background:**

In individuals with schizophrenia, inflammation is associated with depression, somatic comorbidity and reduced quality of life. Physical exercise is known to reduce inflammation in other populations, but we have only limited knowledge in the field of schizophrenia. We assessed inflammatory markers in plasma samples from individuals with schizophrenia participating in an exercise intervention randomized controlled trial. We hypothesized that (i) physical exercise would reduce levels of inflammatory markers and (ii) elevated inflammatory status at baseline would be associated with improvement in cardiorespiratory fitness (CRF) following intervention.

**Method:**

Eighty-two individuals with schizophrenia were randomized to a 12-week intervention of either high-intensity interval training (HIIT, *n* = 43) or active video gaming (AVG, *n* = 39). Participants were assessed at baseline, post intervention and four months later. The associations between exercise and the inflammatory markers soluble urokinase plasminogen activator receptor, c-reactive protein, tumor necrosis factor (TNF), soluble TNF receptor 1 and interleukin 6 (IL-6) were estimated using linear mixed effect models for repeated measures. For estimating associations between baseline inflammation and change in CRF, we used linear regression models.

**Results:**

Our main findings were (i) TNF and IL-6 increased during the intervention period for both groups. Other inflammatory markers did not change during the exercise intervention period; (ii) baseline inflammatory status did not influence change in CRF during intervention, except for a positive association between baseline IL-6 levels and improvements of CRF to post intervention for both groups.

**Conclusion:**

In our study, HIIT and AVG for 12-weeks had no reducing effect on inflammatory markers. Patients with high baseline IL-6 levels had a positive change in CRF during intervention. In order to increase our knowledge regarding association between inflammatory markers and exercise in individuals with schizophrenia, larger studies with more frequent and longer exercise bout duration are warranted.

## 1. Introduction

Schizophrenia continues to be one of the most costly and debilitating mental disorders and is associated with somatic comorbidity and a reduced life-expectancy of 13–15 years (1, 2). During the last decade, increased and altered inflammatory activity has been observed in subgroups of individuals with schizophrenia, triggering questions of associations between inflammation and etiology, symptom subgroups, physical health, prognosis and therapeutic targeting of the disease (3–5).

Inflammation is considered a cause of non-communicable diseases, such as cardiovascular disease (CVD) and type II diabetes, and is associated with schizophrenia, depression, reduced quality of life and reduced motivation (4, 6–9). Thus, inflammation may represent a possible link between schizophrenia and increased risk of somatic and psychiatric comorbidity. Inflammation has also been associated with treatment resistance to antipsychotic drugs (10, 11), and thus poorer psychiatric outcome. A recent meta-analysis of adjunctive anti-inflammatory medication in psychotic disorders found some effect on overall psychopathology, with a stronger effect in schizophrenia compared to other psychotic disorders (12), suggesting that reduction in inflammation may improve general outcomes. Thus, interventions that can dampen the inflammatory status are of high interest.

C-reactive protein (CRP), tumor necrosis factor (TNF), soluble TNF receptor 1 (sTNFR1) and interleukin-6 (IL-6) are among the most frequently investigated inflammatory markers in the field of severe mental illness and exercise research. Increased levels of these markers in schizophrenia have been demonstrated with correlations to symptom severity and cognitive measures (3, 5, 13–15). In the past decade, soluble urokinase plasminogen activator receptor (suPAR) has gained interest as a marker for chronic low-grade inflammation (LGI). Recently, increased levels of suPAR have been demonstrated in individuals with schizophrenia (16, 17), while a smaller study showed no difference compared to healthy controls (18). Notably, suPAR levels correlated with depressive symptoms in individuals with and without schizophrenia, making suPAR-reducing interventions interesting to pursue (6, 19–21).

Physical exercise is associated with a complicated and not fully elucidated interplay between pro-and anti-inflammatory factors. An increase in both pro-and anti-inflammatory factors can be observed in the minutes and hours following an exercise bout. This interplay may be a part of the adaptive mechanisms leading to reduced inflammation following long term exercise (22–24). Low physical activity level is associated with LGI (25, 26) and individuals with schizophrenia display significantly reduced physical activity and cardiorespiratory fitness (CRF) (27–29). Thus, physical exercise in individuals with schizophrenia may have the potential to reduce inflammation and in turn improve somatic and mental health. Studies exploring the effect of physical exercise on LGI in individuals with schizophrenia have been scarce and findings are conflicting. After 8 weeks of exercise in a small, non-randomized, non-blinded study, Heggelund and co-workers found a non-significant CRP decrease in the HIIT-group compared to no change in the comparison group (30). In a later lifestyle modification study in obese, non-diabetic individuals with schizophrenia, Kuo and co-workers were not able to detect significant decrease of either CRP, TNF or IL-6 after 10 weeks of intervention. However, exercise was not extensive (1 h per week), the sample was small and there was no control-group (31). Following these studies, Lavratti and colleagues found a significant decrease in levels of IL-6 and interferon-γ, but not in interleukin 4 (IL-4) after a 12 week exercise program. The study sample was small and there was no control-group (32). In comparison, the current study was an exercise intervention randomized controlled trial (RCT) with a larger sample size and supervised physical activity with a strict protocol examining both commonly used markers of inflammation and the novel suPAR.

We hypothesized that (i) physical exercise would reduce levels of inflammatory markers in individuals with schizophrenia and (ii) enhanced inflammatory status at baseline would be associated with improvement in cardiorespiratory fitness (CRF) following intervention. To explore this, we measured suPAR, CRP, TNF, sTNFR1 and IL-6 in plasma obtained before and after a 12-week exercise intervention RCT in individuals with schizophrenia.

## 2. Methods and materials

The current study is a part of the Effect of Physical Activity on Psychosis Study (EPHAPS), approved by the Regional Committees for Medical and Health Research Ethics (trial number 2014/372), and is examining LGI and factors influencing LGI in a physical exercise intervention RCT with observer-blinded parallel group design (33). The current sample has been studied as a part of a larger cross-sectional sample (6, 16), and in an RCT setting with a physical exercise intervention investigating effect on CRF, cognition and symptoms (34–36).

### 2.1. Participants and randomization

Eighty-two participants were recruited from the two outpatient clinics of Vestfold Hospital Trust in South-Eastern Norway, and participation was based on informed and written consent. Participants were recruited from August 2014 through May 2017. The main inclusion criterion was fulfilling the Diagnostic and Statistical Manual of Mental Disorders (DSM) criteria for schizophrenia spectrum disorders (5^th^ edition), confirmed by the Structured Clinical Interview for DSM-IV [SCID-I (37)]. Additional inclusion criteria were 18–67 years of age and the ability to understand and speak a Scandinavian language. Exclusion criteria were comorbid diagnosis of intellectual disability, pregnancy or medical conditions incompatible with physical exercise. For the current study we also excluded data susceptible to severe infections from participants with CRP >20 (38) due to possible ongoing severe infection at that given time point. However, data from these participants were not excluded at other time points (baseline *n* = 1, post intervention *n* = 1, follow-up *n* = 2).

Participants were recruited by staff dedicated to supervising interventions. A remote study coordinator organized the random allocation to high-intensity interval training (HIIT) or active video gaming (AVG), HIIT (*n* = 43) or AVG (*n* = 39). Participants were stratified on an expected median VO_2peak_, based on results from a feasibility study carried out prior to the EPHAPS project, to ensure similar baseline distribution of VO_2peak_ in the two groups and varied stratification blocks ensured the unpredictability of the treatment assignment (33). The interventions did not interfere with treatment as usual.

### 2.2. Interventions

The HIIT intervention activity was treadmill walking/running following a standardized program of 5 min warm-up, 4 times 4 min high intensity intervals [85%–95% of maximum heart rate (HR_max_)] alternating with 3 min recovery phases of moderate intensity (~70% HR_max_) subsiding with a 5 minute cool-down. The comparison AVG-group, played computerized interactive sport simulation games (Nintendo-Wii sport) in 45 min sessions, and could choose from bowling, golf or tennis simulation. AVG was intended to control for social interaction and time spent with other participants and staff. However, it could be considered to be a low intensity physical activity (39), and, thus, this study compares high and low intensity exercise. We did not measure heart rate during the AVG activity. Both interventions were applied twice a week for 12 weeks in groups of 2–3 participants, remote from assessment sites, and continuously supervised by an intervention team (sport and mental health educated employees trained to supervise both interventions).

### 2.3. Assessments

Trained clinicians, reliability rated on the Structured Clinical Interview for DSM 5 (SCID) and positive and negative syndrome scale (PANSS) (University of California Los Angeles) and blinded for group allocation, obtained medical data and psychometric and physical measures through interviews and medical records. Symptoms and function were measured by PANSS (40, 41), the Calgary depression scale for schizophrenia (CDSS) (42) and the global assessment of functioning scale (GAF) (43) split into the symptoms (GAF-S) and functions (GAF-F) scales to improve psychometric properties (44).

CRF by maximal oxygen uptake (VO_2max_) was assessed through a maximum exercise test on a treadmill (Woodway, Würzburg, Germany), by physical activity professionals. For more detailed information see Andersen et al. (34). We used a modified Balke protocol (45). Measuring respiratory exchange ratio (RER) a value of 1 or more confirmed maximum physical effort by the participants. However, as participants may struggle to reach a RER of 1, we report and implement VO_2peak_ (maximal VO_2_ reached in the test regardless of RER value). As an indirect measure of maximal workload, maximal treadmill speed and inclination were also registered. All assessments and sampling were carried out at baseline (within a two week period before intervention), post intervention (within two weeks of the last intervention-session) and four months later at follow-up (within a two week period) following the same protocols.

#### 2.3.1. Blood sampling and analyses

Fasting blood samples were collected in the morning, and plasma extracted and frozen to −80 degrees Celsius within two hours of sampling. SuPAR, CRP and sTNFR1 were measured in duplicate using a commercially available enzyme-immunoassay (RnDSystems, Stillwater, MN, United States) in a 384-well format using the combination of a SELMA (Jena, Germany) pipetting robot and a BioTek (Winooski, VT, United States) dispenser/washer. Absorption was read at 450 nm with wavelength correction set to 540 nm using an ELISA plate reader (Bio-Rad, Hercules, CA, United States). Intra- and inter-assay coefficients of variation for suPAR, CRP and sTNFR1 were <10%. Detection limits were reported at >40 pg/mL for suPAR, CRP and sTNFR1. IL-6 and TNF were measured in duplicate using a commercially available human high sensitivity T cell magnetic bead panel assay Milliplex (Merck KGaA, Darmstadt, Germany, Millipore Corporation, Billerica, MA, United States). Detection was performed with a Luminex 200 (Luminex, Austin, TX, United States). Intra- and inter-assay coefficients of variation were <5% and <20% for IL-6 and <5% and <15% for TNF, respectively, and detection limits for IL6 was 0.11 pg/mL and 0.16 pg/mL for TNF.

### 2.4. Attrition and protocol violation

Intention-to-treat analyses included all randomized participants and original group assignments. Attendance to sessions in the HIIT group was median 18/mean 15.7 [standard deviation (SD) 7.3] and median 20/mean 18.3 (SD 4.6) in the AVG group, with 24 being the maximum number of sessions. At post intervention and at follow-up 71 (HIIT *n* = 34/AVG *n* = 37) and 57 (HIIT *n* = 29/AVG *n* = 28) individuals were assessed, yielding an attrition rate of 13% at post-intervention, cumulating to 31% at study end. Eight participants in the HIIT group and two in the AVG-group violated the protocol but were allowed to complete the study. In the HIIT group and the AVG group, 25 and 28 participants respectively, were considered per-protocol compliant study completers. For further information on drop out reasons and type of protocol violation, see the EPHAPS consort diagram (35). There were no adverse effects or harm reported during the RCT including the four months follow-up period.

### 2.5. Statistical analyses

Descriptive statistics are presented by frequency and proportion for categorical variables, and by mean, standard deviation (SD) and confidence intervals (CI) for continuous variables. We excluded data sensitive to large ongoing processes in the body (e.g., acute infection, injury or inflammation for other reasons) causing CRP levels to exceed 20 mg/L at that given time point. Following this procedure the inflammatory markers had an approximately normal distribution. However, there were a few outliers (>3 * interquartile range). For these we applied the winsorizing method (46) and truncated the extreme value with the next high value in the dataset in order to include them in the analysis. Correlation analyses were conducted to study the relationship between inflammatory markers, CRF and maximal treadmill speed and inclination. Pearson’s *r* was reported when distribution was normal and Spearman’s rho when distribution was non-normal (e.g., treadmill speed and inclination).

Linear mixed effect models were used to investigate whether levels of inflammatory markers differed over time between the intervention groups. This allows all individuals with at least one measure to be included in the analyses, regardless of missing data at some of the time points. A random factor for participants was included to take into account the variability within individuals over time. An autoregressive correlation matrix was included due to the three categorical time-points. The fixed factors included in the model were group (HIIT/AVG), time, group*time. Margins plots are presented to illustrate the results.

The main analyses were performed in the intention-to-treat sample, and in the dataset where extreme outliers were truncated. To evaluate the robustness of the findings, we also performed a set of supplementary analyses using the per-protocol subset. Additionally, secondary analysis using linear regression were performed to explore the associations between baseline inflammatory markers and change in VO_2peak_ uptake from baseline to post intervention. Group, the inflammatory marker at baseline, and an interaction-term between the two were included as variables. Graphically, scatter plots with smoothed lines are presented. Findings consistently statistically significant (*p* < 0.05) through analysis are emphasized. All statistical analyses were performed in SPSS version 28 and Stata SE version 16.

## 3. Results

Baseline characteristics for the 82 participants included in the study are presented in Table 1. For all clinical and sociodemographic variables, there were no significant differences between the HIIT and AVG groups. Four participants had CRP >20 mg/L at one time point, and data potentially sensitive to inflammatory processes increasing CRP beyond that level were removed from analyses at that given time point.

**Table 1:** Observed demographic and clinical baseline characteristics for participants by randomization group.

Variable	High-intensity interval training (HIIT) (*n* = 43)				Active video gaming (AVG) (*n* = 39)			
	*n*	Mean (SD)	95% CI	%	*n*	Mean (SD)	95% CI	%
*Demographic variables*								
Age (years)	43	36.6 (14.3)	32.2–41.0		39	37.5 (13.8)	33.0–42.0	
Male sex	43			60.5	39			61.5
*Psychopathology*								
GAF function	43	42.2 (7.4)	40.0–44.5		37	45.9 (7.9)	43.3–48.5	
GAF symptoms	43	41.6 (7.6)	39.2–43.9		37	44.8 (8.1)	42.1–47.4	
Duration of illness (years)	36	12.9 (10.3)	9.5–16.4		35	14.6 (12.5)	10.3–18.9	
PANSS positive	42	15.7 (5.1)	14.1–17.3		38	14.2 (5.0)	12.6–15.8	
PANSS negative	42	19.3 (7.2)	17.1–21.6		37	16.9 (6.5)	14.8–19.1	
PANSS general	42	33.4 (7.7)	31.0–35.8		38	32.0 (8.0)	29.3–34.6	
PANSS total	42	68.5 (16.9)	63.2–73.7		37	63.2 (15.3)	58.1–68.3	
CDSS	43	3.5 (3.5)	2.4–4.5		38	3.2 (3.6)	2.0–4.4	
*Lifestyle factors*								
Body mass index (kg/m^2^)	43	29.8 (5.8)	28.0–31.6		39	29.4 (6.4)	27.3–31.4	
Tobacco smoking^a^	43			55.8	39			59.0
*Inflammatory markers*								
suPAR (ng/mL)	41	1.7 (0.4)	1.6–1.8		37	1.6 (0.4)	1.5–1.8	
CRP (mg/L)	41	2.5 (1.5)	2.0–3.0		37	2.3 (1.6)	1.8–2.8	
TNF (pg/L)	42	7.9 (2.9)	7.0–8.8		38	8.1 (3.3)	7.0–9.2	
sTNFR1(ng/mL)	41	1.7 (0.6)	1.5–1.9		37	1.8 (0.6)	1.6–2.0	
IL-6 (pg/mL)	42	3.0 (1.8)	2.5–3.6		38	3.0 (1.6)	2.5–3.5	
*Maximum exercise test*								
VO_2peak_ (L/min)^b^	42	29.9 (11.5)	26.3–33.5		38	29.8 (10.7)	26.3–33.4	
VO_2max_ (L/min)^c^	33	32.2 (11.3)	28.3–36.1		26	31.7 (10.9)	27.3–36.1	
Maximal speed (km/h)^b^	43	4.5 (1.2)	4.1–4.9		38	4.6 (1.1)	4.3–5.0	
Maximal inclination (%)^b^	43	15.9 (3.5)	14.8–16.9		38	15.9 (4.2)	14.5–17.3	

SD, standard deviation; CI, confidence interval; GAF, global assessment of functioning (0–100); PANSS, positive and negative syndrome scale [score ranges: positive (7–49), negative (7–49), general (16–112), total (30–210)]; CDSS, Calgary depression scale for schizophrenia (0–27); suPAR, soluble urokinase plasminogen activator receptor; CRP, C-reactive protein; TNF, tumor necrosis factor; sTNFR1, soluble tumor necrosis factor receptor-1IL-6, interleukin 6; VO_2_max, maximum oxygen uptake; RER, respiratory exchange ratio.

a Tobacco smoking classified as current.

b VO_2peak_, maximal speed and maximal inclination were assessed using a treadmill-based maximum exercise test (27, 45).

c VO_2max_, maximal oxygen uptake when including only individuals with a RER ≥ 1 used to indicate that the participants are working at the limits of their cardiorespiratory system and that the VO_2max_ test is valid.

All baseline inflammatory markers showed inverse correlations with VO_2peak_ and/or max speed and/or max inclination at baseline. However, this was most pronounced and highly significant for suPAR (*r* −0.4 to −0.5, *p* < 0.001), CRP (*r* −0.3 to −0.4, *p* < 0.01) and sTNFR1 (*r* −0.3 to −0.4, *p* < 0.05) as these three markers significantly correlated with VO_2max_, treadmill max speed and max inclination (see Table 2).

**Table 2:** Correlation between inflammatory markers and measures of cardiorespiratory fitness and workload at baseline.

	VO_2peak_ (L/min)^a^		Maximal speed (km/h)^a^		Maximal inclination (%)^a^	
	Pearson’s r	*p*-value	Spearman’s rho	*p*-value	Spearman’s rho	*p*-value
suPAR (ng/mL)	**−0.51**	**<0.001**	**−0.42**	**<0.001**	**−0.43**	**<0.001**
CRP (mg/L)	**−0.36**	**<0.01**	**−0.39**	**<0.001**	**−0.31**	**<0.01**
TNF (pg/L)	−0.21	0.07	**−0.23**	**0.04**	−0.15	0.19
sTNFR1 (ng/mL)	**−0.26**	**0.03**	**−0.29**	**0.01**	**−0.36**	**0.01**
IL-6 (pg/mL)	**−0.28**	**0.01**	−0.20	0.08	**−0.24**	**0.03**

suPAR, soluble urokinase plasminogen activator receptor; CRP, C-reactive protein; TNF, Tumor necrosis factor IL-6, interleukin 6; sTNFR1, soluble tumor necrosis factor receptor-1; VO_2peak_ maximal oxygen uptake achieved in test. Bold script indicates significance according to preset *p*-value of <0.05.

a VO_2peak_, maximal speed and maximal inclination were assessed using a treadmill-based maximum exercise test protocol (27, 45).

### 3.1. Alteration in inflammatory markers following the 12-week exercise program

**Figure 1:**
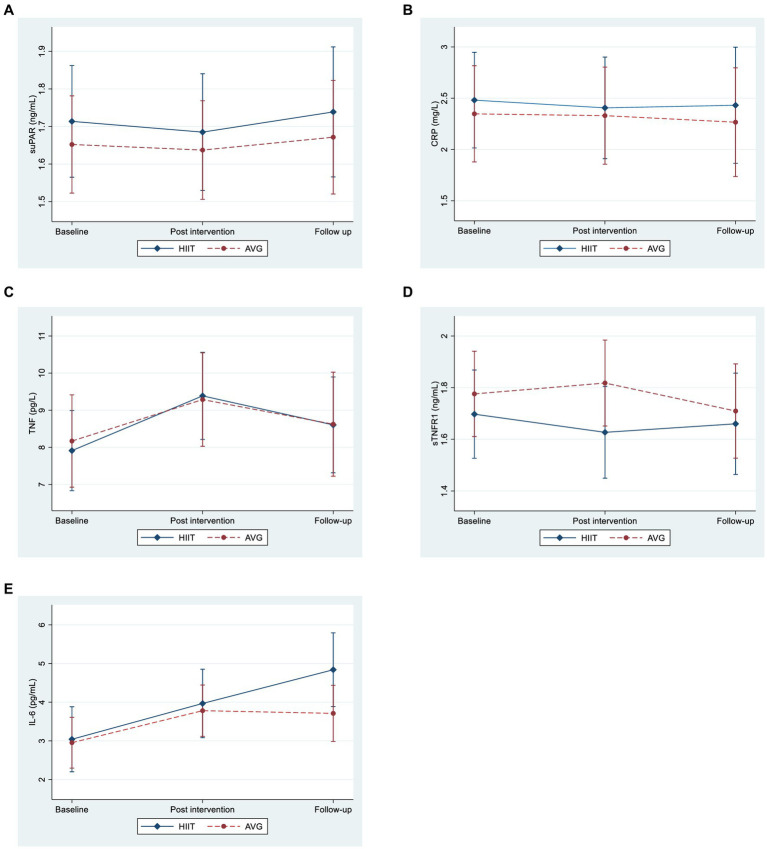
Margin plots showing the time development of inflammation marker levels in the high intensity training (HIIT) and active video gaming (AVG) group with 95% confidence interval in a schizophrenia sample, *n* = 79 for these analyses due to missing data (*n* = 3). Time was measured at baseline (before intervention), post intervention (within 2 weeks of the intervention-session) and 4 months later at follow-up. **(A)** suPAR, soluble urokinase plasminogen activating receptor; **(B)** CRP, C-reactive protein; **(C)** TNF, tumor necrosis factor; **(D)** sTNFR1, soluble tumor necrosis factor receptor; **(E)** 1IL-6, interleukin 6.

We first evaluated the effect of exercise intervention on the temporal course of inflammatory markers. We did not observe any differences in the levels of suPAR, CRP and TNFR1 over time or between the intervention groups at any time-point (HIIT vs. AVG) (Figure 1 and Supplementary Table S1). For TNF and IL-6, there were no significant differences between the intervention groups, but the markers increased from baseline to post intervention (i.e., at 12 weeks) for both groups (TNF 1.27, 95% CI 0.49, 2.05, *p* = 0.001, IL-6 0.86, 95% CI 0.47, 1.26, *p* < 0.001). Further, IL-6 continued to increase at the four month follow-up (IL-6 1.14, 95% CI 0.59, 1.70, *p* < 0.001), while TNF returned to baseline levels. Regardless of group affiliation, we found no significant association between number of intervention sessions and change in any of the inflammatory markers or change in VO_2peak_ from baseline to post intervention (data not shown).

### 3.2. Associations between inflammatory markers at baseline and changes in CRF following the 12-week physical exercise program

**Figure 2:**
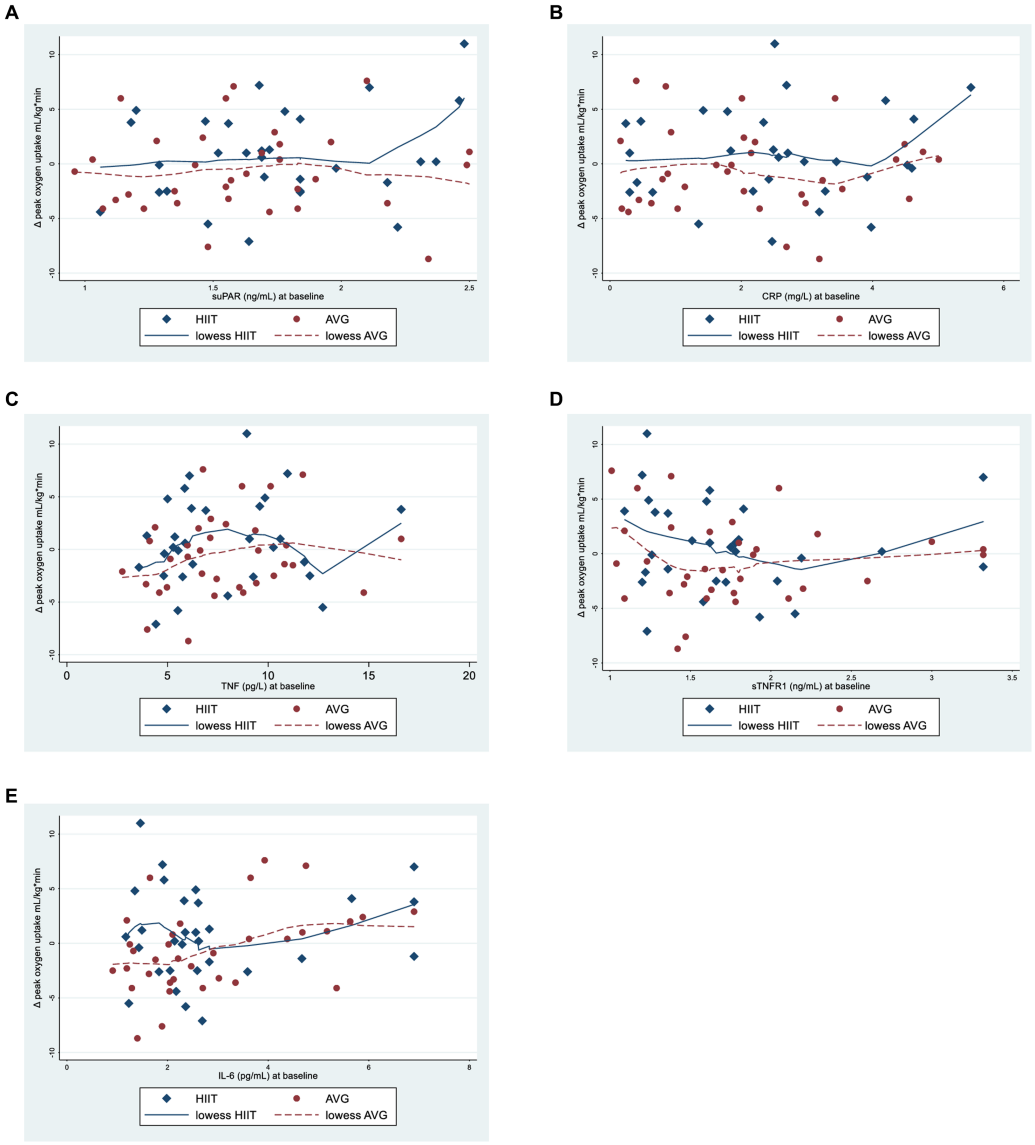
Scatter plots with smoothed lowess lines showing the relationship between inflammatory maker levels at baseline and change in peak oxygen uptake [mL/(kg*min)] in the high intensity interval training (HIIT) and active video gaming (AVG) group from baseline to post treatment. **(A)** suPAR, soluble urokinase plasminogen activating receptor; **(B)** CRP, C-reactive protein; **(C)** TNF, tumor necrosis factor; **(D)** sTNRF1, soluble tumor necrosis factor receptor; **(E)** 1IL-6, interleukin 6.

In our secondary analyses, we assessed if CRF at post intervention was associated with baseline inflammatory levels, and if this association differed between intervention groups. The association between inflammatory markers at baseline and change in CRF (VO_2peak_) from baseline to post intervention showed no significant differences between HIIT and AVG (Figure 2), i.e., the interaction terms between inflammatory markers at baseline and group were all non-significant (Supplementary Table S2). However, the level of IL-6 at baseline was positively associated with improvement in VO_2peak_ (0.67, 95% CI 0.06, 1.27, *p* = 0.03) regardless of group allocation.

### 3.3. Supplementary analyses

The supplementary analyses restricted to the per-protocol subsample, yielded similar results as the main analyses when investigating differences between groups over time for all the inflammatory markers. Since no interactions between group and time were observed, the interactions terms were omitted from the analyses (models both with and without the interaction terms are shown in Supplementary Table S1). Furthermore, the results for the per-protocol subsample were similar as the main analysis when studying the importance of inflammatory baseline levels on the CRF change over time (Supplementary Table S2).

### 3.4. *Post-hoc* analyses

Intrigued by the increase in TNF and IL-6, we performed a set of *post-hoc* analyses, trying to understand this phenomenon. Three different mixed effect models were performed for each of the two inflammatory markers (TNF and IL-6). In addition to the time variable (baseline/post intervention/follow-up) we adjusted for (1) sex and age, (2) time-dependent body mass index (BMI) values and (3) physical activity competency of staff in the model. Due to the small sample size no additional adjustment were performed. The TNF and IL-6 changes observed over time in the main analyses were not significantly affected by the adjustments by sex and age, BMI and physical activity competency of the staff (data not shown).

## 4. Discussion

In a naturalistic sample of 82 outpatients diagnosed with schizophrenia, all the inflammatory markers, but in particular suPAR and CRP, were inversely correlated with CRF at baseline. Contraire to our hypothesis we found no significant differences between HIIT and AVG on temporal levels of inflammatory markers or any generalized distinct reduction in inflammation levels following the exercise intervention. In contrast, TNF and IL-6 increased regardless of intervention group. Levels of inflammatory markers at baseline were not indicative of improvement in CRF following intervention, with the exception of IL-6 levels which were associated with improvements of CRF in both groups. Consequently, our data do not support the hypothesis that physical exercise reduces general immune activation, at least as reflected by our choice of upstream inflammatory markers, interventions, or time points of measure.

The inverse correlations between CRF and inflammatory markers are in line with previous research (26, 47–50) implying that physical activity could lead to a reduction in levels of inflammatory markers as reported by others (51, 52). In contrast, we observed no reduction in inflammatory markers. However, many participants were not able to adhere completely to the HIIT protocol, and some had lower intensity than anticipated and/or failed to conduct most sessions (34). This might have inflicted on our results. Also, it is possible that the HIIT intervention was too limited in bout duration, number of weekly interventions and duration of the exercise period to dampen immune activation. The chosen time points of measurement could also have affected the results, as blood was drawn >24 h after the last exercise bout, and we were not able to capture potential changes in the acute dynamic regulation of the inflammatory markers. Nonetheless, the results in the current study are in line with other studies in individuals with schizophrenia (30, 31), while Lavratti et al. demonstrated a decrease in inflammatory markers following a 90-day exercise intervention program (32). However, comparisons across studies are challenged by different exercise interventions, low sample sizes and non-blinded and non-randomized designs. Regarding suPAR, our results are in line with two studies on suPAR and chronic exercise, showing no reduction in suPAR levels (53, 54).

The increases in IL-6 and TNF from baseline to post intervention were unexpected due to the time of sampling (>24 h from the last exercise bout), and are thus difficult to explain. Had the blood samples been taken in close proximity to the last exercise bout we would have expected an increase in inflammatory markers (55). Overtraining occurs when an athlete does not recover after repetitive intense training and has been associated with an increase in inflammatory markers (56). However, with only two HIIT bouts per week, overtraining is not likely to be the cause of the observed increases in TNF and IL-6. Beside, similar increases were observed in the AVG group. IL-6 is also produced locally in working skeletal muscle, and enhanced release during exercise could potentially account for the increase during intervention. Furthermore, muscle-derived IL-6 may possess anti-inflammatory effects (57, 58), thus whether the IL-6 increase is an expression of pro- or anti-inflammatory effects is uncertain. Similar to the current study healthy individuals participating at a 3-month yoga retreat also showed an increase in pro-inflammatory cytokines (e.g., TNF and IL-6), which was unexpected. However they also observed an increase in the anti-inflammatory interleukin 10 (IL-10), which we did not measure in our study (59). Our finding of increased inflammation by TNF and IL-6, following a 3 month exercise program could thus be due to an interplay between pro-and inflammatory factors responding to acute bouts of exercise, adaption to several acute bouts of exercise over time, as well as the bodily changes following chronic exercise.

In a prior study we found no significant change in CRF as a result of the interventions, however the HIIT group had significant improvement in maximal treadmill inclination and speed (measured at specific points during intervention) (34). As this was unexpected we hypothesized that baseline LGI level could be one of the reasons for lack of effect on CRF in the HIIT group. High baseline levels of IL-6 were associated with better exercise effect on CRF for both the HIIT and the AVG group, regardless of type of interventions prescribed. Senescence is associated with unhealthy lifestyle and ageing and causes increased expression of inflammatory markers (e.g., IL-6) (60). Therefore individuals with high levels of inflammatory markers could be those with less healthy lifestyle and thus with more potential for improvements through exercise. However, with a wide confidence interval reflecting the small sample size and the heterogeneity within the individuals recruited to the study, this finding can only be regarded as suggestive of an effect.

There are relatively few exercise studies in individuals with schizophrenia as recruiting and retaining participants is demanding. Studies of the immune system in this type of setting have rarely been conducted and results are conflicting. The above-mentioned heterogeneity within schizophrenia could very well be one of the reasons for the controversy in the field. This further highlights the need for larger studies of inflammation in individuals with schizophrenia and of interventions aimed at reducing inflammation. Our study was relatively large, observer blinded, had strict exercise and test protocols, and exercise sessions were supervised as opposed to prior studies on exercise and inflammation in individuals with schizophrenia. The naturalistic sample of motivated outpatients with schizophrenia suggests high generalizability of the findings. However, the results must be interpreted with caution as there are several limitations to the study. Even though the study was large in this context, it is likely to be underpowered. Despite the relatively large size compared to prior studies in this field, the sample is also too small to conduct concurrent adjustments for possible confounders likely to be of importance [e.g., age, sex, current tobacco smoking, diet, fat mass and BMI (61)] or to stratify into subgroups, e.g., inflammation in the exercise responsive sub-population within our sample. The study participants ranged from newly diagnosed with schizophrenia to living with the illness for decades and they were relatively stable. The relatively small sample size made stratification into subgroups based on duration of illness or current state (e.g., exacerbation vs. stable) difficult. A recent study conducted after inflammatory markers were analyzed in our study, showed that the method we used to analyze suPAR was inferior to another method (62). Also, our control group participated in a low intensity activity and an additional control-group performing no activity would have been a strength. A longer lasting intervention would have been preferable, as a clear change in CRF could have led to firmer conclusions on the relationship between inflammation and exercise in individuals with schizophrenia. In a prior study of this sample we found no change in positive or negative symptoms, but a reduction in depressive symptoms following the HIIT intervention (36). A larger study exploring the relationships between inflammatory markers and depressive symptoms in the context of a HIIT intervention in individuals with schizophrenia could yield important results.

## 5. Conclusion

In our study, HIIT and AVG for 12-weeks had no lowering effect on plasma levels of inflammatory markers. Patients with high IL-6 levels at baseline experienced an increase in CRF during intervention. There is a need for methodologically robust studies in the field of schizophrenia, inflammation and exercise treatment, investigating dose-response and comparing different exercise-modes.

## Data availability statement

The datasets presented in this article are not readily available because data are sensitive in nature and as such the availability is restricted and regulated by Norwegian Laws and EC laws (GDPR). Requests to access the datasets should be directed to JAE (PI), uxjogh@siv.no.

## Ethics statement

The studies involving human participants were reviewed and approved by Regional Committees for Medical and Health Research Ethics (trial number 2014/372), rekportalen.no. The patients/participants provided their written informed consent to participate in this study.

## Author contributions

JAE (PI) and JE (co-PI) initiated the conception and design of the EPHAPS project. TB, MF, JN, TV, and JM contributed to the idea and design of this study. TB, GB-K, TH, JAE, JE, and EA contributed to data collection and preparation of the database. TB, RF, and MF contributed to the analyses and main interpretation of the data. TU contributed to the laboratory analyses. TB wrote the first draft of the manuscript. All authors made critical revisions and partially contributed to interpretation of data in the writing process and approved the final version of the manuscript.

## Funding

The EPHAPS study received funding from South-Eastern Norway Regional Health Authorities (Helse Sør-Øst) (TB), Vestfold Hospital Trust, Norwegian Extra Foundation for Health and Rehabilitation through EXTRA funds, Norwegian Research Network in Severe Mental Illness (NORSMI), NORMENT/K.G. Jebsen Centre for Psychosis Research, Josef and Haldis Andresen’s foundation, Torgeir Lindvik’s Trust and Civitan International.

## Conflict of interest

The authors declare that the research was conducted in the absence of any commercial or financial relationships that could be construed as a potential conflict of interest.

## Publisher’s note

All claims expressed in this article are solely those of the authors and do not necessarily represent those of their affiliated organizations, or those of the publisher, the editors and the reviewers. Any product that may be evaluated in this article, or claim that may be made by its manufacturer, is not guaranteed or endorsed by the publisher.

## References

[1] Charlson FJ Ferrari AJ Santomauro DF Diminic S Stockings E Scott JG. Global epidemiology and burden of schizophrenia: findings from the global burden of disease study 2016. Schizophr Bull. (2018) 44:1195–203. doi: 10.1093/schbul/sby058, PMID: 29762765 PMC6192504

[2] Hjorthoj C Sturup AE McGrath JJ Nordentoft M. Years of potential life lost and life expectancy in schizophrenia: a systematic review and meta-analysis. Lancet Psychiatry. (2017) 4:295–301. doi: 10.1016/S2215-0366(17)30078-0, PMID: 28237639

[3] Bishop JR Zhang L Lizano P. Inflammation subtypes and translating inflammation-related genetic findings in schizophrenia and related psychoses: a perspective on pathways for treatment stratification and novel therapies. Harv Rev Psychiatry. (2022) 30:59–70. doi: 10.1097/HRP.0000000000000321, PMID: 34995036 PMC8746916

[4] Goldsmith DR Rapaport MH Miller BJ. A meta-analysis of blood cytokine network alterations in psychiatric patients: comparisons between schizophrenia, bipolar disorder and depression. Mol Psychiatry. (2016) 21:1696–709. doi: 10.1038/mp.2016.3, PMID: 26903267 PMC6056174

[5] Kroken RA Sommer IE Steen VM Dieset I Johnsen E. Constructing the immune signature of schizophrenia for clinical use and research; an integrative review translating descriptives into diagnostics. Front Psychiatry. (2018) 9:753. doi: 10.3389/fpsyt.2018.00753 30766494 PMC6365449

[6] Bigseth TT Engh JA Egeland J Andersen E Andreassen OA Bang-Kittilsen G. Exploring low grade inflammation by soluble urokinase plasminogen activator receptor levels in schizophrenia: a sex-dependent association with depressive symptoms. BMC Psychiatry. (2021) 21:527. doi: 10.1186/s12888-021-03522-6, PMID: 34702245 PMC8547032

[7] Conley RR Ascher-Svanum H Zhu B Faries DE Kinon BJ. The burden of depressive symptoms in the long-term treatment of patients with schizophrenia. Schizophr Res. (2007) 90:186–97. doi: 10.1016/j.schres.2006.09.027, PMID: 17110087 PMC1937504

[8] Faugere M Micoulaud-Franchi JA Alessandrini M Richieri R Faget-Agius C Auquier P. Quality of life is associated with chronic inflammation in schizophrenia: a cross-sectional study. Sci Rep. (2015) 5:10793. doi: 10.1038/srep10793 26041435 PMC4455112

[9] Pena-Oyarzun D Bravo-Sagua R Diaz-Vega A Aleman L Chiong M Garcia L. Autophagy and oxidative stress in non-communicable diseases: a matter of the inflammatory state? Free Radic Biol Med. (2018) 124:61–78. doi: 10.1016/j.freeradbiomed.2018.05.084, PMID: 29859344

[10] Labonte C Zhand N Park A Harvey PD. Complete blood count inflammatory markers in treatment-resistant schizophrenia: evidence of association between treatment responsiveness and levels of inflammation. Psychiatry Res. (2022) 308:114382. doi: 10.1016/j.psychres.2021.114382, PMID: 34995832

[11] Mondelli V Ciufolini S Belvederi Murri M Bonaccorso S Di Forti M Giordano A. Cortisol and inflammatory biomarkers predict poor treatment response in first episode psychosis. Schizophr Bull. (2015) 41:1162–70. doi: 10.1093/schbul/sbv028, PMID: 25829375 PMC4535637

[12] Jeppesen R Christensen RHB Pedersen EMJ Nordentoft M Hjorthoj C Kohler-Forsberg O. Efficacy and safety of anti-inflammatory agents in treatment of psychotic disorders—a comprehensive systematic review and meta-analysis. Brain Behav Immun. (2020) 90:364–80. doi: 10.1016/j.bbi.2020.08.028, PMID: 32890697

[13] North HF Bruggemann J Cropley V Swaminathan V Sundram S Lenroot R. Increased peripheral inflammation in schizophrenia is associated with worse cognitive performance and related cortical thickness reductions. Eur Arch Psychiatry Clin Neurosci. (2021) 271:595–607. doi: 10.1007/s00406-021-01237-z, PMID: 33760971

[14] Orsolini L Sarchione F Vellante F Fornaro M Matarazzo I Martinotti G. Protein-C reactive as biomarker predictor of schizophrenia phases of illness? A systematic review. Curr Neuropharmacol. (2018) 16:583–606. doi: 10.2174/1570159X16666180119144538, PMID: 29357805 PMC5997872

[15] Momtazmanesh S Zare-Shahabadi A Rezaei N. Cytokine alterations in schizophrenia: an updated review. Front Psychiatry. (2019) 10:892. doi: 10.3389/fpsyt.2019.00892, PMID: 31908647 PMC6915198

[16] Bigseth TT Fredriksen M Egeland J Andersen E Andreassen OA Bang-Kittilsen G. Elevated levels of soluble urokinase plasminogen activator receptor as a low-grade inflammation marker in schizophrenia: a case-control study. Schizophr Res. (2021) 228:190–2. doi: 10.1016/j.schres.2020.11.051, PMID: 33450603

[17] Nielsen J Roge R Pristed SG Viuff AG Ullum H Thorner LW. Soluble urokinase-type plasminogen activator receptor levels in patients with schizophrenia. Schizophr Bull. (2015) 41:764–71. doi: 10.1093/schbul/sbu118, PMID: 25154621 PMC4393680

[18] Genc A Kalelioglu T Karamustafalioglu N Tasdemir A Genc ES Akkus M. Serum soluble urokinase-type plasminogen activator receptor levels in male patients with acute exacerbation of schizophrenia. Psychiatry Res. (2016) 236:179–81. doi: 10.1016/j.psychres.2016.01.018, PMID: 26774189

[19] Haastrup E Grau K Eugen-Olsen J Thorball C Kessing LV Ullum H. Soluble urokinase plasminogen activator receptor as a marker for use of antidepressants. PLoS One. (2014) 9:e110555. doi: 10.1371/journal.pone.0110555, PMID: 25329298 PMC4203805

[20] Latham RM Kieling C Arseneault L Kohrt BA Moffitt TE Rasmussen LJH. Longitudinal associations between adolescents’ individualised risk for depression and inflammation in a UK cohort study. Brain Behav Immun. (2022) 101:78–83. doi: 10.1016/j.bbi.2021.12.027, PMID: 34990745 PMC8906711

[21] Ventorp F Gustafsson A Traskman-Bendz L Westrin A Ljunggren L. Increased soluble urokinase-type plasminogen activator receptor (suPAR) levels in plasma of suicide attempters. PLoS One. (2015) 10:e0140052. doi: 10.1371/journal.pone.0140052, PMID: 26451727 PMC4599802

[22] Cronin O Keohane DM Molloy MG Shanahan F. The effect of exercise interventions on inflammatory biomarkers in healthy, physically inactive subjects: a systematic review. QJM. (2017) 110:629–37. doi: 10.1093/qjmed/hcx091, PMID: 28472518

[23] Fischer CP. Interleukin-6 in acute exercise and training: what is the biological relevance? Exerc Immunol Rev. (2006) 12:6–33. PMID: 17201070

[24] Ostrowski K Rohde T Asp S Schjerling P Pedersen BK. Pro- and anti-inflammatory cytokine balance in strenuous exercise in humans. J Physiol. (1999) 515:287–91. doi: 10.1111/j.1469-7793.1999.287ad.x, PMID: 9925898 PMC2269132

[25] Pedersen BK. The diseasome of physical inactivity—and the role of myokines in muscle-fat cross talk. J Physiol. (2009) 587:5559–68. doi: 10.1113/jphysiol.2009.179515, PMID: 19752112 PMC2805368

[26] Beavers KM Brinkley TE Nicklas BJ. Effect of exercise training on chronic inflammation. Clin Chim Acta. (2010) 411:785–93. doi: 10.1016/j.cca.2010.02.069, PMID: 20188719 PMC3629815

[27] Andersen E Holmen TL Egeland J Martinsen EW Bigseth TT Bang-Kittilsen G. Physical activity pattern and cardiorespiratory fitness in individuals with schizophrenia compared with a population-based sample. Schizophr Res. (2018) 201:98–104. doi: 10.1016/j.schres.2018.05.038, PMID: 29861267

[28] Stubbs B Williams J Gaughran F Craig T. How sedentary are people with psychosis? A systematic review and meta-analysis. Schizophr Res. (2016) 171:103–9. doi: 10.1016/j.schres.2016.01.034, PMID: 26805414

[29] Vancampfort D Rosenbaum S Probst M Soundy A Mitchell AJ De Hert M. Promotion of cardiorespiratory fitness in schizophrenia: a clinical overview and meta-analysis. Acta Psychiatr Scand. (2015) 132:131–43. doi: 10.1111/acps.12407, PMID: 25740655

[30] Heggelund J Nilsberg GE Hoff J Morken G Helgerud J. Effects of high aerobic intensity training in patients with schizophrenia: a controlled trial. Nord J Psychiatry. (2011) 65:269–75. doi: 10.3109/08039488.2011.560278, PMID: 21332297 PMC3169036

[31] Kuo FC Lee CH Hsieh CH Kuo P Chen YC Hung YJ. Lifestyle modification and behavior therapy effectively reduce body weight and increase serum level of brain-derived neurotrophic factor in obese non-diabetic patients with schizophrenia. Psychiatry Res. (2013) 209:150–4. doi: 10.1016/j.psychres.2012.11.020, PMID: 23219101

[32] Lavratti C Dorneles G Pochmann D Peres A Bard A de Lima SL. Exercise-induced modulation of histone H4 acetylation status and cytokines levels in patients with schizophrenia. Physiol Behav. (2017) 168:84–90. doi: 10.1016/j.physbeh.2016.10.021, PMID: 27810494

[33] Engh JA Andersen E Holmen TL Martinsen EW Mordal J Morken G. Effects of high-intensity aerobic exercise on psychotic symptoms and neurocognition in outpatients with schizophrenia: study protocol for a randomized controlled trial. Trials. (2015) 16:557. doi: 10.1186/s13063-015-1094-2, PMID: 26646670 PMC4672547

[34] Andersen E Bang-Kittilsen G Bigseth TT Egeland J Holmen TL Martinsen EW. Effect of high-intensity interval training on cardiorespiratory fitness, physical activity and body composition in people with schizophrenia: a randomized controlled trial. BMC Psychiatry. (2020) 20:425. doi: 10.1186/s12888-020-02827-2, PMID: 32854688 PMC7457274

[35] Bang-Kittilsen G Egeland J Holmen TL Bigseth TT Andersen E Mordal J. High-intensity interval training and active video gaming improve neurocognition in schizophrenia: a randomized controlled trial. Eur Arch Psychiatry Clin Neurosci. (2021) 271:339–53. doi: 10.1007/s00406-020-01200-4, PMID: 33156372

[36] Bang-Kittilsen G Engh JA Holst R Holmen TL Bigseth TT Andersen E. High-intensity interval training may reduce depressive symptoms in individuals with schizophrenia, putatively through improved VO_2_max: a randomized controlled trial. Front Psychiatry. (2022) 13:921689. doi: 10.3389/fpsyt.2022.921689, PMID: 36003983 PMC9394183

[37] Spitzer RL Williams JB Gibbon M First MB. The structured clinical interview for DSM-III-R (SCID): I. History, rationale, and description. Arch Gen Psychiatry. (1992) 49:624–9. doi: 10.1001/archpsyc.1992.01820080032005 1637252

[38] Hoseth EZ Ueland T Dieset I Birnbaum R Shin JH Kleinman JE. A study of TNF pathway activation in schizophrenia and bipolar disorder in plasma and brain tissue. Schizophr Bull. (2017) 43:881–90. doi: 10.1093/schbul/sbw183, PMID: 28049760 PMC5515106

[39] Peng W Lin JH Crouse J. Is playing exergames really exercising? A meta-analysis of energy expenditure in active video games. Cyberpsychol Behav Soc Netw. (2011) 14:681–8. doi: 10.1089/cyber.2010.0578, PMID: 21668370

[40] Kay SR Fiszbein A Opler LA. The positive and negative syndrome scale (PANSS) for schizophrenia. Schizophr Bull. (1987) 13:261–76. doi: 10.1093/schbul/13.2.261 3616518

[41] Wallwork RS Fortgang R Hashimoto R Weinberger DR Dickinson D. Searching for a consensus five-factor model of the positive and negative syndrome scale for schizophrenia. Schizophr Res. (2012) 137:246–50. doi: 10.1016/j.schres.2012.01.031, PMID: 22356801 PMC3351536

[42] Addington D Addington J Maticka-Tyndale E. Assessing depression in schizophrenia: the Calgary depression scale. Br J Psychiatry Suppl. (1993) 22:39–44. 8110442

[43] Endicott J Spitzer RL Fleiss JL Cohen J. The global assessment scale. A procedure for measuring overall severity of psychiatric disturbance. Arch Gen Psychiatry. (1976) 33:766–71. doi: 10.1001/archpsyc.1976.01770060086012 938196

[44] Pedersen G Karterud S. The symptom and function dimensions of the global assessment of functioning (GAF) scale. Compr Psychiatry. (2012) 53:292–8. doi: 10.1016/j.comppsych.2011.04.007 21632038

[45] Balke B Ware RW. An experimental study of physical fitness of air force personnel. U S Armed Forces Med J. (1959) 10:675–88. PMID: 13659732

[46] Field A. Discovering Statistics Using IBM SPSS Statistics. London, England: SAGE Publications (2013).

[47] Elosua R Bartali B Ordovas JM Corsi AM Lauretani F Ferrucci L. Association between physical activity, physical performance, and inflammatory biomarkers in an elderly population: the InCHIANTI study. J Gerontol A Biol Sci Med Sci. (2005) 60:760–7. doi: 10.1093/gerona/60.6.760, PMID: 15983180

[48] Ishikawa H Izumiya Y Shibata A Ichikawa Y Yamaguchi T Yamaguchi Y. Soluble urokinase-type plasminogen activator receptor represents exercise tolerance and predicts adverse cardiac events in patients with heart failure. Heart Vessel. (2020) 35:681–8. doi: 10.1007/s00380-019-01538-3, PMID: 31741050

[49] Wedell-Neergaard AS Krogh-Madsen R Petersen GL Hansen AM Pedersen BK Lund R. Cardiorespiratory fitness and the metabolic syndrome: roles of inflammation and abdominal obesity. PLoS One. (2018) 13:e0194991. doi: 10.1371/journal.pone.0194991, PMID: 29590212 PMC5874061

[50] Madssen E Skaug EA Wisloff U Ellingsen O Videm V. Inflammation is strongly associated with cardiorespiratory fitness, sex, BMI, and the metabolic syndrome in a self-reported healthy population: HUNT3 fitness study. Mayo Clin Proc. (2019) 94:803–10. doi: 10.1016/j.mayocp.2018.08.040, PMID: 30935704

[51] Zheng G Qiu P Xia R Lin H Ye B Tao J. Effect of aerobic exercise on inflammatory markers in healthy middle-aged and older adults: a systematic review and meta-analysis of randomized controlled trials. Front Aging Neurosci. (2019) 11:98. doi: 10.3389/fnagi.2019.00098, PMID: 31080412 PMC6497785

[52] Gleeson M Bishop NC Stensel DJ Lindley MR Mastana SS Nimmo MA. The anti-inflammatory effects of exercise: mechanisms and implications for the prevention and treatment of disease. Nat Rev Immunol. (2011) 11:607–15. doi: 10.1038/nri3041, PMID: 21818123

[53] Rohde C Polcwiartek C Andersen E Vang T Nielsen J. Effect of a physical activity intervention on suPAR levels: a randomized controlled trial. J Sci Med Sport. (2018) 21:286–90. doi: 10.1016/j.jsams.2017.06.018, PMID: 28728886

[54] Sponder M Lichtenauer M Wernly B Paar V Hoppe U Emich M. Serum heart-type fatty acid-binding protein decreases and soluble isoform of suppression of tumorigenicity 2 increases significantly by long-term physical activity. J Investig Med. (2019) 67:833–40. doi: 10.1136/jim-2018-000913, PMID: 30593542

[55] Ostermann S Herbsleb M Schulz S Donath L Berger S Eisentrager D. Exercise reveals the interrelation of physical fitness, inflammatory response, psychopathology, and autonomic function in patients with schizophrenia. Schizophr Bull. (2013) 39:1139–49. doi: 10.1093/schbul/sbs085, PMID: 22966149 PMC3756770

[56] Docherty S Harley R McAuley JJ Crowe LAN Pedret C Kirwan PD. The effect of exercise on cytokines: implications for musculoskeletal health: a narrative review. BMC Sports Sci Med Rehabil. (2022) 14:5. doi: 10.1186/s13102-022-00397-2, PMID: 34991697 PMC8740100

[57] Nara H Watanabe R. Anti-inflammatory effect of muscle-derived Interleukin-6 and its involvement in lipid metabolism. Int J Mol Sci. (2021) 22:9889. doi: 10.3390/ijms22189889, PMID: 34576053 PMC8471880

[58] Petersen AM Pedersen BK. The anti-inflammatory effect of exercise. J Appl Physiol. (2005) 98:1154–62. doi: 10.1152/japplphysiol.00164.2004 15772055

[59] Cahn BR Goodman MS Peterson CT Maturi R Mills PJ. Yoga, meditation and mind-body health: increased BDNF, cortisol awakening response, and altered inflammatory marker expression after a 3-month yoga and meditation retreat. Front Hum Neurosci. (2017) 11:315. doi: 10.3389/fnhum.2017.00315, PMID: 28694775 PMC5483482

[60] Liu Z Wu KKL Jiang X Xu A Cheng KKY. The role of adipose tissue senescence in obesity- and ageing-related metabolic disorders. Clin Sci. (2020) 134:315–30. doi: 10.1042/CS20190966, PMID: 31998947

[61] Haupt TH Kallemose T Ladelund S Rasmussen LJ Thorball CW Andersen O. Risk factors associated with serum levels of the inflammatory biomarker soluble urokinase plasminogen activator receptor in a general population. Biomark Insights. (2014) 9:91–100. doi: 10.4137/BMI.S19876, PMID: 25574132 PMC4269129

[62] Abraham AG Xu Y Roem JL Greenberg JH Weidemann DK Sabbisetti VS. Variability in CKD biomarker studies: soluble urokinase plasminogen activator receptor (suPAR) and kidney disease progression in the chronic kidney disease in children (CKiD) study. Kidney Med. (2021) 3:712–721.e1. doi: 10.1016/j.xkme.2021.04.007, PMID: 34693253 PMC8515077

