# Supplementary material for: Alterations in inflammatory markers after a 12-week exercise program in individuals with schizophrenia—a randomized controlled trial
Source: Front Psychiatry. 2023 May 11;14:1175171. doi: 10.3389/fpsyt.2023.1175171 (PMC10231033; doi:10.3389/fpsyt.2023.1175171)
Supplement: Supplementary file 1 [file Table_1.DOCX]

| **Supplementary table S1**  **Inflammatory markers over time in the two intervention groups, including per protocol analyses** | | | | | | | | |
| --- | --- | --- | --- | --- | --- | --- | --- | --- |
| Inflammatory marker | Intention to treat analyses | | | | Per protocol analyses | | | |
|  | Variable | Estimate | 95% CI | p | Variable | Estimate | 95% CI | p |
| suPAR (ng/ml)^a^ | HIIT group | 0.06 | -0.14, 0.26 | 0.54 | HIIT group | 0.07 | -0.20, 0.35 | 0.61 |
|  | Time: post intervention | -0.01 | -0.13, 0.10 | 0.79 | Time: post intervention | -0.04 | -0.18, 0.09 | 0.55 |
|  | Time: follow-up | 0.02 | -0.14, 0.18 | 0.81 | Time: follow-up | -0.01 | -0.19, 0.18 | 0.95 |
|  | HIIT group x post intervention | -0.01 | -0.17, 0.14 | 0.86 | HIIT group x post intervention | 0.02 | -0.17, 0.21 | 0.86 |
|  | HIIT group x follow-up | 0.01 | -0.22, 0.23 | 0.96 | HIIT group x follow-up | 0.05 | -0.21, 0.32 | 0.69 |
| suPAR (ng/ml)^b^ | HIIT group | 0.06 | -0.12, 0.24 | 0.51 | HIIT group | 0.09 | -0.15, 0.34 | 0.45 |
|  | Time: post intervention | -0.02 | -0.10, 0.05 | 0.57 | Time: post intervention | -0.03 | -0.13, 0.06 | 0.48 |
|  | Time: follow-up | 0.02 | -0.09, 0.13 | 0.69 | Time: follow-up | 0.02 | -0.11, 0.15 | 0.76 |
| CRP  (mg/L)^a^ | HIIT group | 0.13 | -0.53, 0.79 | 0.69 | HIIT group | 0.43 | -0.38, 1.24 | 0.30 |
|  | Time: post intervention | -0.02 | -0.36, 0.32 | 0.92 | Time: post intervention | -0.04 | -0.40, 0.32 | 0.84 |
|  | Time: follow-up | -0.08 | -0.58, 0.42 | 0.75 | Time: follow-up | -0.12 | -0.62, 0.37 | 0.63 |
|  | HIIT group x post intervention | -0.06 | -0.59, 0.47 | 0.83 | HIIT group x post intervention | -0.15 | -0.74, 0.44 | 0.61 |
|  | HIIT group x follow-up | 0.03 | -0.73, 0.80 | 0.94 | HIIT group x follow-up | -0.10 | -0.91, 0.72 | 0.82 |
| CRP  (mg/L)^b^ | HIIT group | 0.13 | -0.46, 0.72 | 0.67 | HIIT group | 0.36 | -0.35, 1.08 | 0.32 |
|  | Time: post intervention | -0.04 | -0.30, 0.22 | 0.76 | Time: post intervention | -0.09 | -0.38, 0.19 | 0.52 |
|  | Time: follow-up | -0.07 | -0.44, 0.31 | 0.73 | Time: follow-up | -0.16 | -0.55, 0.23 | 0.42 |
| TNF  (pg/L)^a^ | HIIT group | -0.26 | -1.91, 1.39 | 0.76 | HIIT group | 0.12 | -1.77, 2.01 | 0.90 |
|  | **Time: post intervention** | **1.12** | **0.08, 2.16** | **0.036** | **Time: post intervention** | **1.15** | **0.05, 2.24** | **0.040** |
|  | Time: follow-up | 0.45 | -1.01, 1.91 | 0.54 | Time: follow-up | 0.62 | -0.77, 2.00 | 0.38 |
|  | HIIT group x post intervention | 0.36 | -1.23, 1.95 | 0.66 | HIIT group x post intervention | 0.26 | -1.61, 2.12 | 0.79 |
|  | HIIT group x follow-up | 0.24 | -1.87, 2.35 | 0.82 | HIIT group x follow-up | 0.04 | -2.27, 2.35 | 0.97 |
| TNF  (pg/L)^b^ | HIIT group | -0.11 | -1.49, 1.28 | 0.88 | HIIT group | 0.19 | -1.29, 1.66 | 0.80 |
|  | **Time: post intervention** | **1.27** | **0.49, 2.05** | **0.001** | **Time: post intervention** | **1.24** | **0.36, 2.11** | **0.006** |
|  | Time: follow-up | 0.57 | -0.48, 1.61 | 0.29 | Time: follow-up | 0.63 | -0.47, 1.73 | 0.26 |
| sTNFR1  (ng/ml)^a^ | HIIT group | -0.08 | -0.32, 0.16 | 0.52 | HIIT group | 0.02 | -0.25, 0.29 | 0.87 |
|  | Time: post intervention | 0.04 | -0.06, 0.15 | 0.43 | Time: post intervention | 0.03 | -0.09, 0.16 | 0.59 |
|  | Time: follow-up | -0.07 | -0.22, 0.09 | 0.41 | Time: follow-up | -0.06 | -0.22, 0.11 | 0.50 |
|  | HIIT group x post intervention | -0.11 | -0.27, 0.04 | 0.16 | HIIT group x post intervention | -0.11 | -0.28, 0.07 | 0.23 |
|  | HIIT group x follow-up | 0.03 | -0.20, 0.26 | 0.81 | HIIT group x follow-up | 0.06 | -0.19, 0.30 | 0.66 |
| sTNFR1  (ng/ml)^b^ | HIIT group | -0.09 | -0.31, 0.13 | 0.42 | HIIT group | 0.02 | -0.22, 0.27 | 0.84 |
|  | Time: post intervention | -0.01 | -0.08, 0.07 | 0.83 | Time: post intervention | -0.02 | -0.11, 0.07 | 0.66 |
|  | Time: follow-up | -0.05 | -0.17, 0.06 | 0.39 | Time: follow-up | -0.03 | -0.16, 0.09 | 0.58 |
| IL-6  (pg/mL)^a^ | HIIT group | 0.09 | -0.98, 1.16 | 0.87 | HIIT group | 0.06 | -1.32, 1.43 | 0.93 |
|  | **Time: post intervention** | **0.83** | **0.34, 1.31** | **0.001** | **Time: post intervention** | **0.61** | **0.08, 1.14** | **0.023** |
|  | **Time: follow-up** | **0.76** | **0.06, 1.45** | **0.033** | Time: follow-up | 0.63 | -0.07, 1.33 | 0.08 |
|  | HIIT group x post intervention | 0.10 | -0.72, 0.92 | 0.81 | HIIT group x post intervention | 0.34 | -0.67, 1.35 | 0.51 |
|  | HIIT group x follow-up | 1.04 | -0.11, 2.19 | 0.08 | HIIT group x follow-up | 1.33 | -0.01, 2.68 | 0.052 |
| IL-6  (pg/mL)^b^ | HIIT group | 0.43 | -0.54, 1.40 | 0.38 | HIIT group | 0.67 | -0.54, 1.88 | 0.28 |
|  | **Time: post intervention** | **0.86** | **0.47, 1.26** | **<0.001** | **Time: post intervention** | **0.70** | **0.25, 1.16** | **0.002** |
|  | **Time: follow-up** | **1.14** | **0.59, 1.70** | **<0.001** | **Time: follow-up** | **0.98** | **038, 1.59** | **0.001** |
| *Note. Estimate = Estimated coefficient; suPAR = Soluble urokinase plasminogen activator receptor; CRP = C-reactive protein; TNF = Tumor necrosis factor; sTNFR1 = Soluble tumor necrosis factor receptor 1; IL-6 = Interleukin 6.*  ***Bold text indicates significance (p<0.05)***  *^a^ Model including group, time and group x time interaction (inspecting differences between the groups (HIIT vs AVG) over time)*  *^b^ Model including time and group (not group x time interaction if this term was insignificant)* | | | | | | | | |
