# Supplementary material for: Alterations in inflammatory markers after a 12-week exercise program in individuals with schizophrenia—a randomized controlled trial
Source: Front Psychiatry. 2023 May 11;14:1175171. doi: 10.3389/fpsyt.2023.1175171 (PMC10231033; doi:10.3389/fpsyt.2023.1175171)
Supplement: Supplementary file 2 [file Table_2.DOCX]

| **Supplementary table S2**  **Change in VO_2peak_ from baseline to post intervention adjusted for baseline levels of inflammatory markers and tested for interaction between level of inflammation at baseline and group (HIIT or AVG) in intention-to-treat and per-protocol** | | | | | | | |
| --- | --- | --- | --- | --- | --- | --- | --- |
|  |  | Intention-to-treat | | | Per-Protocol | | |
| Inflammatory marker | Variables | Estimate | 95%CI | p | Estimate | 95%CI | p |
| suPAR  (ng/mL)^a^ | Baseline suPAR | 0.15 | -3.38, 3.69 | 0.93 | 0.41 | -3.47, 4.29 | 0.83 |
|  | HIIT group | -2.91 | -12.00-6.16 | 0.52 | -6.73 | -17.07, 3.60 | 0.20 |
|  | Interaction term | 2.47 | -2.77, 7.71 | 0.35 | 4.82 | -1.09, 10.73 | 0.11 |
| suPAR  (ng/mL)^b^ | Baseline suPAR | 1.28 | -1.33, 3.88 | 0.33 | 2.49 | 0.50, 5.47 | 0.10 |
|  | HIIT group | 1.25 | -0.82, 3.31 | 0.23 | 1.44 | -1.11, 4.00 | 0.26 |
| CRP  (mg/L)^a^ | Baseline CRP | -0.12 | -1.11, 0.87 | 0.81 | -0.11 | -1.33, 1.1 | 0.85 |
|  | HIIT group | 0.43 | -3.53, 4.38 | 0.83 | -1.24 | -6.57, 4.08 | 0.64 |
|  | Interaction term | 0.41 | -1.03, 1.85 | 0.57 | 1.10 | -0.74, 2.94 | 0.23 |
| CRP  (mg/L)^b^ | Baseline CRP | 0.08 | -0.64, 0.79 | 0.83 | 0.37 | -0.54, 1.29 | 0.42 |
|  | HIIT group | 1.38 | -0.68, 3.45 | 0.19 | 1.54 | -1.11, 4.18) | 0.25 |
| TNF  (pg/L)^a^ | Baseline TNF | 0.26 | -0.19, 0.71 | 0.26 | 0.23 | -0.44, 0.89 | 0.49 |
|  | HIIT group | 2.01 | -3.46, 7.48 | 0.47 | 1.90 | -5.37, 9.16 | 0.60 |
|  | Interaction term | -0.08 | -0.73, 0.58 | 0.81 | -0.04 | -0.93, 0.85 | 0.93 |
| TNF  (pg/L)^b^ | Baseline TNF | 0.22 | -0.10, 0.54 | 0.18 | 0.21 | -0.23, 0.64 | 0.35 |
|  | HIIT group | 1.40 | -0.59, 3.39 | 0.17 | 1.59 | -0.95, 4.12 | 0.22 |
| sTNFR1  (ng/mL)^a^ | Baseline sTNFR1 | -0.16 | -2.59, 2.27 | 0.90 | -1.36 | -5.12, 2.40 | 0.47 |
|  | HIIT group | 2.22 | -4.41, 8.85 | 0.51 | -1.10 | -10.43, 8.24 | 0.81 |
|  | Interaction term | -0.48 | -4.10, 3.14 | 0.79 | 1.69 | -3.58, 6.97 | 0.52 |
| sTNFR1  (ng/mL)^b^ | Baseline sTNFR1 | -0.37 | -2.16, 1.42 | 0.68 | -0.60 | -3.00, 1.81 | 0.60 |
|  | HIIT group | 1.39 | -0.66, 3.44 | 0.18 | 1.78 | -0.83, 4.39 | 0.18 |
| IL-6  (pg/mL)^a^ | Baseline IL-6 | **1.00** | **0.16, 1.84** | **0.020** | 1.02 | -0.10, 2.14 | 0.07 |
|  | HIIT group | 3.39 | -0.59, 7.36 | 0.09 | 2.60 | -2.44, 7.63 | 0.30 |
|  | Interaction term | -0.69 | -1.90, 0.52 | 0.26 | -0.35 | -1.92, 1.21 | 0.65 |
| IL-6  (pg/mL)^b^ | Baseline IL-6 | **0.67** | **0.06, 1.27** | **0.031** | **0.84** | **0.07, 1.61** | **0.03** |
|  | HIIT group | 1.41 | -0.54, 3.35 | 0.15 | 1.60 | -0.83, 4.03 | 0.19 |
| *Note. Estimate = Estimated coefficient; suPAR = Soluble urokinase plasminogen activator receptor; CRP = C-reactive protein; TNF = Tumor necrosis factor; sTNFR1 = Soluble tumor necrosis factor receptor 1; IL-6 = Interleukin 6.* ***Bold text indicates significance (p<0.05)***  *^a^Model including baseline inflammatory marker, group (*HIIT vs AVG*) and interaction term between the baseline inflammatory marker and group.*  *^b^Model including baseline inflammatory marker and group (*HIIT vs AVG*) only (i.e. no interaction term included).* | | | | | | | |
